# Potential Neurochemical and Neuroendocrine Effects of Social Distancing Amidst the COVID-19 Pandemic

**DOI:** 10.3389/fendo.2020.582288

**Published:** 2020-10-08

**Authors:** Limin Wang, Ghulam Nabi, Tonghe Zhang, Yuefeng Wu, Dongming Li

**Affiliations:** Key Laboratory of Animal Physiology, Biochemistry and Molecular Biology of Hebei Province, College of Life Sciences, Hebei Normal University, Shijiazhuang, China

**Keywords:** coronavirus disease 2019, social isolation, psychological health, neurohormones, neurotransmitters, neurotrophins

## Introduction

Coronavirus disease 2019 (COVID-19) was first identified in Wuhan, China, in December 2019. It was then declared a public health emergency of international concern on 30^th^ January 2020. Due to the exceptionally high transmissibility and robust infectiousness of the underlying virus [severe acute respiratory syndrome coronavirus 2 (SARS-CoV-2)] (1), it was finally declared a pandemic on 11^th^ March 2020 (2). As of August 1^st^ 2020, SARS-CoV-2 had spread widely and rapidly in more than 200 countries worldwide, causing 17 million confirmed cases and a staggering death toll of 0.68 million (3).

Since no vaccine is far available for containment of this virus, thus unprecedented large-scale quarantine measures are one of the best ways to prevent the chain of events leading to transmission of SARS-CoV-2. In many countries, mandatory requirements for social isolation, including stay-at-home orders, banning social contacts, canceling public events, and closing mass transit systems have been ordered by governments (4, 5). Although large-scale quarantines are effective at preventing transmission of the virus, they can be extremely unpleasant for those enduring them, leading to long-term social isolation and severe impacts on psychological health (4, 6, 7). Moreover, the lack of basic supplies as a result of long-term social isolation and the economic losses resulting from isolation, which have exacerbated people’s feelings of panic, thus increasing the risk of developing psychological disorders (8). Here, we summarize some of the detrimental psychological, neuroendocrine, and neurochemical changes induced by the chronic social isolation of the COVID-19 pandemic and provide concrete recommendations for alleviating these changes.

## Psychological Effects of Chronic Social Isolation

In social animals, positive social interactions are fundamental for increasing cognitive ability, promoting brain development, and maintaining mental and physical health, ultimately contributing to survival and reproduction (9, 10). Long-term interruption of these social bonds is a predisposing factor for many neuropsychiatric diseases (11, 12). Numerous studies have shown that short-term isolation (acute alone or repeated acute) in humans and other non-human mammals can dramatically modify social behavior and can disturb the neurochemical and neuroendocrine system and brain functioning (13), with long-term isolation (human: more than 10 days; rodents: 2 to 9 weeks) leading to more serious outcomes (14–17).

Stress due to social isolation accounts for one of the most common sources of chronic stress in humans. Previous studies have shown that chronic social isolation correlates with a higher risk of morbidity and mortality (18). Chronic social isolation has also been shown to be a leading cause of many psychological disorders, including schizophrenia (19, 20), depression, anxiety, social withdrawal (16), and learning deficits (21). These psychological abnormalities may be caused by stress-induced neurochemical and neuroendocrine alterations (4). The latest statistics suggest that a long period of social isolation during the COVID-19 outbreak may lead to an increase in mental illness among different populations. For example, recent evidences shown that pandemics increased the prevalence of post-traumatic stress disorder (PTSD) (4), as well as feeling of loneliness (4), irritability (1), boredom (22), phobias (23), and social dysfunction (23) after social isolation. Therefore, controlling the length of quarantine to what is scientifically reasonable, given the known duration of incubation periods would minimize the impact on human mental health.

## Neuroendocrine Effects of Chronic Social Isolation

The brain is a vital organ that is vulnerable to influence by the external environment. Regulation of social behavior and emotion depends on complex neural circuits within the brain (24). Previous studies have shown that prolonged periods of social isolation (over 10 days in human; 2 to 9 weeks in rodents) can exert profound effects on the brain, which, in turn, can affect behavior and mood in humans (14) and non-human mammals (25–27). Neurohormones and neurotransmitters have long been implicated as important mediators of behavior and psychological action-related neural circuits in both human beings and animals (28–31). These mediators exert their modulatory effects on brain circuits by activating G protein-coupled receptors, which, in turn, allow for changes in neuronal excitability, thereby altering behavior in invertebrate [nematode and drosophila: (28, 30)] and non-human vertebrate [rodent: (29)]. Moreover, hormones such as corticotropin-releasing hormone (CRH), arginine vasopressin (AVP), and adrenocorticotropic hormone (ACTH) involving in regulation of gene transcription could potentially influence neuronal excitability and neuronal survival, finally coordinate the neuroendocrine response to stress, and also modulate behaviors (32, 33). It is important to recognize that chronic stress-induced abnormalities in the neurochemical and neuroendocrine system may predispose to public panic and mental health disorders during the COVID-19 pandemic.

Exposure to social isolation stress induces a variety of endocrine changes, and dysregulation of the neuroimmune-endocrine system has been shown to be one vital mechanism for the development of psychological disorders (34). Studies have shown that deprivation of social interaction can stimulate the hypothalamic-pituitary-adrenal (HPA) axis through increased secretion of stress hormones (glucocorticoids), which are involved in metabolic and immunological regulation (26). Additionally, social isolation can inhibit the release of the social hormones, oxytocin and vasopressin, in the hypothalamus, which modulate social behaviors in humans (35, 36) and non-human animals (26, 37). In fact, intranasal delivery of vasopressin and oxytocin has been shown to compensate for the negative effects of social isolation in a previous study by Lieberwirth and Wang (36).

## Neurochemical Effects of Chronic Social Isolation

Furthermore, social isolation stress has also been shown to alter levels of neurotransmitters and receptor sensitivities in many regions of the central nervous system (CNS) (38, 39). Interruption of these neurotransmitter pathways has been shown to play a major role in the development of psychological disorders in socially isolated animals. Previous studies have shown that social isolation can suppress the release of several neurotransmitters, including dopamine, serotonin, adrenaline, gamma-aminobutyric acid (GABA), and glutamate, which reduces “happiness” and increases psychological distress or mental illness in rats or mice (38–42). If these neurotransmitter system perturbations occur over the long term, they can have direct and indirect detrimental consequences on metabolism, immunity, anxiety, depression, and post-traumatic stress disorder (26).

Social isolation stress can also alter neurotrophin levels. Brain-derived neurotrophic factor (BDNF) is a member of the neurotrophin family of growth factors, which are key regulators of synaptogenesis, neuronal plasticity, and adult neurogenesis (43, 44), creating potentially important links between stress and mental illness (45). Evidence has indicated that chronic social isolation is associated with decreased BDNF mRNA and protein expression in rodents (2 to 9 weeks) (46–49) and decreased BDNF levels in human peripheral blood (the exact time length of “chronic” is not specified (45, 50). If the COVID-19 pandemic is not effectively controlled, necessitating chronic social isolation, reductions in neuronal plasticity and adult neurogenesis may occur by decreasing levels of nerve growth factor (NGF) and BDNF, eventually leading to changes in brain function and structural plasticity (51). Studies also have shown that chronic social isolation stress is associated with reductions in the volume of the hippocampus and that chronic stress can modulate the volumes of both the amygdala and frontal cortex (52).

## Discussion

For many countries, the COVID-19 pandemic may necessitate long-term and wide-ranging social isolation. This social isolation will not only change the reaction norms of daily activities, but may also create dramatic adverse psychological effects, including loss of freedom, isolation from friends and family, and feelings of loneliness and uncertainty. Unfortunately, chronic stress-induced neurochemical and neuroendocrine disorders are often neglected until they become psychological illness. Given that a mandatory quarantine of confirmed and suspected cases is the best way to avoid spread of COVID-19, a series of measures are needed to avoid the negative physiological and psychological effects induced by chronic social isolation, including potential neurochemical and neuroendocrine system disorders. First, national public health emergency systems should provide fundamental and explicit guidelines for prevention of mental illness induced by mandatory social distancing and thereby mitigate physiological and psychological stressors. These guidelines should include recommendations for positive social interactions and maintenance/development of social supports, including online communication with relatives and friends (53). Second, timely positive social interactions and specialized psychological interventions should be geared at people most at risk for experiencing physiological and mental stress during this pandemic, including healthcare workers, confirmed COVID-19 patients undergoing treatment, and patients with preexisting psychiatric illnesses (54, 55). Third, the use of digital technologies by employers should also be promoted to boost public confidence and happiness and to decrease anxiety by reducing workload burdens and increasing efficiency (56, 57).

In conclusion, both governments and citizens must recognize the important neurochemical and neuroendocrine changes induced by the chronic social isolation stress of the COVID-19 pandemic to help prevent potential psychological and mental illnesses that are often invisible and easy to neglect.

## Author Contributions

LW: literature searched and wrote the original draft. GN: literature searched and wrote the original draft. TZ: manuscript revised. DL and YW: study designed. DL: wrote the final manuscript. All authors contributed to the article and approved the submitted version.

## Funding

This work was supported by the National Natural Science Foundation of China (NSFC; 31672292) to DL and the Natural Science Foundation of China of Hebei Province (C2020205005) and China Postdoctoral Science Foundation (2020M670685) to LW.

## Conflict of Interest

The authors declare that the research was conducted in the absence of any commercial or financial relationships that could be construed as a potential conflict of interest.
